# Nano-Delivery Systems for Essential Oils in Chitosan-Based Biopolymer Packaging: Structure-Function Relationships and Active-Intelligent Applications

**DOI:** 10.3390/foods15132395

**Published:** 2026-07-06

**Authors:** Qin Liu, Hanahati Kuerbanjiang, Xiaofeng Ren, You Shi, Lixin Kang, Yuxuan Liu, Qiufang Liang, Mingming Zhong, Yufan Sun, Xinyu Chen, Wenjing Zhu, Arif Rashid

**Affiliations:** 1 School of Food and Biological Engineering, Jiangsu University, Zhenjiang 212013, China; liuqin496043732@163.com (Q.L.); dllyx1997@163.com (Y.L.); lqf@ujs.edu.cn (Q.L.); mingming.zhong@ujs.edu.cn (M.Z.); yufan.sun@ujs.edu.cn (Y.S.); 2 School of Agricultural Engineering, Jiangsu University, Zhenjiang 212013, China; zwj0410@foxmail.com; 3 Forestry Science Research Institute in Ili Kazakh Autonomous Prefecture, Forestry Science and Technology Building, No. 259 Feijichang Road, Yining 835000, China; gameking0524@163.com; 4 School of Food Science and Technology, Jiangnan University, Wuxi 214122, China; kanglx0320@163.com; 5 School of Optoelectronic Engineering, Changzhou Institute of Technology, No. 666 Liaohe Road, Changzhou 213002, China; chenxiny@czust.edu.cn; 6 College of Food Science and Engineering, Shandong Agricultural University, Tai’an 271018, China; arif_ktk007@hotmail.com

**Keywords:** chitosan, essential oils, nano-delivery systems, active food packaging, interfacial regulation, controlled release, food preservation

## Abstract

Although chitosan (CS)- and essential oil (EO)-based packaging systems have been widely reviewed, a focused synthesis connecting nano-delivery design with interfacial regulation, film-network evolution, release behavior, and preservation performance in real food systems remains lacking. This review addresses that gap by examining CS-based nano-delivery systems for EOs in active food packaging, with an emphasis on how carrier design and multiscale organization govern functional performance. Major delivery strategies, including nanoemulsions, nanoparticles, nanogels, Pickering emulsions, nanofibrous systems, and nanocomposites, are discussed in relation to EO stabilization, dispersion uniformity, and controlled release. Their effects on film microstructure, mechanical and barrier properties, thermal stability, optical behavior, and antimicrobial and antioxidant activities are further evaluated alongside preservation outcomes in fruits, vegetables, dairy products, meat, and aquatic products. Particular attention is given to structure-function relationships across the carrier, interface, and film-network levels, and to the distinction between established active-packaging functions and emerging smart-packaging applications. Current challenges include EO compositional variability, limited cross-study comparability, sensory constraints, migration and regulatory concerns, and insufficiently scalable fabrication routes. Future work should prioritize mechanism-informed interfacial design, standardized evaluation frameworks, food-specific release-preservation correlations, and scalable green manufacturing.

## 1. Introduction

The extensive use of petroleum-based plastics in food packaging has raised serious environmental concerns and intensified the demand for biodegradable, non-toxic, and sustainable alternatives. In this context, bioactive films based on chitosan (CS), a naturally derived polysaccharide, and plant-derived essential oils (EOs) have attracted increasing interest as candidate materials for food packaging applications [1,2]. EOs are natural volatile compounds with antimicrobial, antioxidant, and flavor-related properties that can suppress microbial growth, retard oxidative deterioration, and extend the shelf life of perishable foods, including fruits, vegetables, dairy products, and meat [3,4]. Because of these multifunctional attributes, EOs have been widely investigated as active agents in food packaging systems [5,6].

Despite these advantages, the direct incorporation of EOs into packaging matrices remains challenging because of their volatility, hydrophobicity, chemical instability, and sensitivity to environmental factors such as light, oxygen, and temperature [5,6]. In addition, excessive or poorly controlled incorporation of EOs may adversely affect the sensory quality of packaged foods. To address these limitations, nanostructuring strategies, including nanoencapsulation, emulsion stabilization, and nanocomposite design, have been developed to improve EOs stability, enhance dispersion homogeneity, and regulate release behavior while maintaining compatibility with different food matrices [4,7]. These approaches provide an effective means of improving the retention, distribution, and sustained functionality of EOs in packaging films.

CS is a particularly attractive carrier material because of its biodegradability, film-forming ability, biocompatibility, and inherent antimicrobial activity [1,2,8]. When combined with EOs through nano-delivery strategies, CS can improve EOs stabilization, modulate film microstructure, and enhance physicochemical and functional properties, including mechanical strength, barrier performance, and bioactivity. These features make CS-EO systems promising for active food packaging and may also support their integration into freshness-indicating or stimuli-responsive packaging systems when combined with suitable responsive components [9,10,11,12].

Although CS-EOs systems have been extensively reviewed, existing reviews have largely focused on encapsulation methodologies, composite film properties, or food preservation applications [3,4,7]. However, systematic comparative analyses are still lacking regarding how different nano-delivery platforms regulate EOs behavior through structural design, and how EOs affect film performance. In addition, the design potential of these systems for smart-packaging applications has rarely been systematically explored.

This review systematically examines six representative CS-based nano-delivery platforms, including nanofibers, nanogels, nanoparticles, nanoemulsions, Pickering emulsions, and nanocomposites. In contrast to previous reviews, this review shifts the analytical focus from how these systems are prepared to how their structures determine performance. Specifically, we discuss the regulatory mechanisms by which each platform modulates the stability, dispersion, and release of EOs, as well as the resulting effects on the physicochemical properties of the films. In addition, the discussion is extended to smart-packaging applications (freshness-indicating and stimuli-responsive controlled-release systems), a perspective that has been less systematically integrated in previous reviews. Finally, this review identifies the key challenges in translating these systems from laboratory research to commercial application, including safety assessment, regulatory acceptance, manufacturing scalability, and consumer trust. Based on the above analysis, this review aims to provide a clear roadmap for the rational design of CS-EOs packaging systems with targeted preservation functions.

## 2. Properties of CS and EOs

### 2.1. Structure and Properties of CS

CS is a semicrystalline polysaccharide derived from the partial deacetylation of chitin and is mainly composed of β-(1→4)-linked D-glucosamine and N-acetyl-D-glucosamine units. Its abundant amino and hydroxyl groups confer high chemical reactivity and enable extensive intermolecular interactions, whereas protonation of amino groups under acidic conditions gives CS its characteristic polycationic nature. These structural attributes largely determine its solubility, chain interactions, and biological behavior, while also providing the molecular basis for its performance in film-forming systems [13,14].

In food-packaging applications, CS is widely valued because it combines film-forming capacity with biodegradability, biocompatibility, and intrinsic antimicrobial activity. Hydrogen bonding and chain entanglement facilitate the formation of continuous films with acceptable structural integrity, while its natural origin and degradability further support its use as a sustainable packaging material [13,15,16]. CS is also more than a passive structural polymer. Its cationic amino groups can interact with negatively charged microbial cell surfaces, disrupt membrane function, and interfere with microbial activity, thereby contributing to antibacterial performance [17]. Taken together, these characteristics make CS a suitable matrix for packaging systems that require both structural support and bioactive functionality.

However, pure CS films still exhibit limitations that may restrict their practical applicability. In particular, their moisture sensitivity and limited thermoplasticity can compromise film stability and barrier performance, especially under humid storage conditions [13,15]. CS should therefore be regarded as a promising platform material whose performance often depends on further modification or integration with complementary active components.

### 2.2. Bioactive Properties and Practical Limitations of EOs

EOs are volatile natural extracts obtained from different parts of aromatic plants, including leaves, flowers, fruits, seeds, bark, and roots [18]. They are complex mixtures of low-molecular-weight compounds such as terpenes, terpenoids, phenolics, aldehydes, ketones, alcohols, and esters [19]. This compositional diversity is closely associated with their broad range of biological activities.

EOs are widely recognized for their antimicrobial and antioxidant properties, both of which are highly relevant to food preservation. Their antimicrobial activity generally involves multiple mechanisms, including disruption of microbial membranes, leakage of intracellular contents, interference with metabolic processes, and inhibition of enzyme activity [20]. This multi-target mode of action is particularly valuable for controlling foodborne spoilage and pathogenic microorganisms and has also attracted increasing attention for its potential against multidrug-resistant foodborne pathogens [21]. In addition, the antioxidant activity of many EOs can retard oxidative deterioration in food systems, thereby contributing to quality maintenance and shelf-life extension [22]. Consistent with this view, nanoencapsulation can further strengthen the functional expression of EOs; for example, Citrus reticulata EO nanocapsules co-stabilized with clove and cinnamon EOs showed enhanced antioxidant activity, antifungal activity, and oxidation stability, highlighting the value of structural protection and compositional synergy in EO-based preservation systems [23].

Despite these advantages, the direct incorporation of free EOs into polymer matrices is associated with several limitations. Their high volatility can lead to substantial losses during processing and storage, while their hydrophobic nature often results in poor dispersion in hydrophilic matrices such as CS [24]. EOs are also sensitive to environmental factors such as oxygen, light, and heat, which may accelerate degradation and reduce their functional efficacy [24]. Moreover, interactions with food components can modify their antimicrobial effectiveness and may also affect sensory attributes, especially when higher EO concentrations are required [25]. As a result, directly incorporated EOs often show uneven distribution, insufficient retention, and uncontrolled release behavior, all of which limit their effectiveness in packaging systems [26].

### 2.3. Rationale for Integrating CS and EOs Through Nano-Delivery Systems

When EOs are incorporated into CS-based films, their behavior within the matrix becomes a key determinant of both material properties and preservation performance. Because CS is hydrophilic, whereas EOs are predominantly hydrophobic and volatile, direct blending often leads to non-uniform distribution of the active phase, partial loss of volatile constituents, and local concentration heterogeneity within the film. These effects can weaken matrix continuity and reduce the stability and persistence of EO-mediated antimicrobial and antioxidant functions.

The performance of CS-EO systems is therefore closely linked to how the active phase is retained, distributed, and released within the material. Insufficient retention may reduce the amount of active compounds available during storage, whereas heterogeneous dispersion may introduce structural irregularities into the film matrix. In addition, rapid or uncontrolled release may shorten the period over which EOs remain functionally effective, creating a mismatch between EO activity and the actual preservation requirements of food systems [26].

Under these conditions, the organization of EOs within the matrix becomes particularly important. When EOs are confined within nanoscale carriers or nanostructured domains, their exposure to environmental stressors can be reduced, their dispersion in the CS matrix can be improved, and their release behavior can be more effectively regulated [20]. Such structural organization allows the active phase to be incorporated into the film more stably and uniformly, thereby improving the consistency and effectiveness of CS-based packaging systems. In this context, nano-delivery systems can be understood as a rational strategy for enhancing the stability, distribution, and functional persistence of EOs within the film matrix.

## 3. Structural Platforms for EO Nano-Delivery in CS-Based Active Food Packaging

Direct incorporation of EOs into CS-based packaging systems rarely provides adequate retention, uniform distribution, or predictable release. Once incorporated into food-contact materials, EOs may be lost prematurely, distributed heterogeneously, or show unstable functional performance, particularly under complex storage conditions and in compositionally diverse food matrices. Under such circumstances, the way EOs are structurally organized within CS-based systems becomes a major determinant of preservation performance [6,27].

Different CS-based nano-delivery platforms regulate EO behavior through distinct structural and interfacial mechanisms. Fibrous networks, crosslinked gel domains, discrete nanoparticles, nanoemulsion droplets, particle-stabilized interfaces, and nanocomposite matrices differ not only in how EOs are incorporated, but also in the degree to which EO stabilization, dispersion, retention, and sustained release can be controlled. These differences ultimately influence which platform is most suitable for a given preservation target, food matrix, or storage environment [6,27].

From this perspective, CS-based EO nano-delivery systems are better understood as structural platforms for active food packaging rather than as simple carriers. Their relevance lies in how material organization, interfacial regulation, and release-related behavior determine EO retention, local availability, and functional expression under food-contact conditions. Accordingly, the following sections examine representative platforms for EO nano-delivery in CS-based active food packaging, namely nanofibers, nanogels, nanoparticles, nanoemulsions, Pickering emulsions, and nanocomposites, with emphasis on how each platform governs EO incorporation, stabilization, dispersion, and release in relation to preservation performance.

### 3.1. Nanofibers

CS-based NFs provide a distinctive platform for EO delivery in active food packaging because their continuous fibrous networks combine high interfacial area with tunable porosity. This architecture facilitates EOs incorporation while creating diffusion pathways that moderate EO loss from the matrix. Compared with discrete particulate carriers, nanofibrous systems are more closely associated with surface-localized protection, making them particularly relevant for solid foods and fresh produce that require prolonged local bioactivity [6,28].

The interaction between CS and EOs exerts a significant influence on the microstructure of the nanofibers. When the CS/poly (vinyl alcohol) mass ratio was 5:5, the encapsulation efficiencies of the prepared nanofibers for lemon, lime, and grapefruit EOs were 52.57%, 56.95%, and 47.76%, respectively. When the CS/poly (vinyl alcohol) ratio was increased to 7:3, the encapsulation efficiencies for the three EOs increased to 85.56%, 87.16%, and 83.95%, respectively. This improvement indicates that higher CS content provides more interaction sites, thereby enhancing the binding efficiency of EOs and leading to better encapsulation [29].

Fibrous morphology further affects EOs release and the persistence of preservation-related activity. The incorporation of eucalyptus EOs into gelatin/CS NFs resulted in a decrease in the diameter of the fibers, as well as alterations in the network organization. Concurrently, there was a modification of air permeability and an enhancement of thermal stability. The CS/gelatin nanofibers loaded with eucalyptus EOs were prepared via electro-blowing, with an average fiber diameter of 260.39 ± 5.05 nm, forming nanofibers free of droplets and bead-like structures [30]. Carvacrol was loaded into gelatin/CS composite NFs membranes via electrospinning. As the carvacrol content increased from 1.0% to 10.0% (*w*/*w*), the nanofiber diameter increased from 0.82 ± 0.17 μm to 0.99 ± 0.14 μm. This is attributed to the decrease in solution conductivity caused by the addition of carvacrol, which led to thicker fiber morphology and larger diameter [31]. The encapsulation efficiency of gelatin/CS composite nanofibers for 1% *w*/*w* carvacrol was as high as 92.85%, but when the carvacrol content was increased to 5% *w*/*w*, the encapsulation efficiency decreased to 36.17 ± 1.35% [31]. This indicates that the loading capacity of CS and gelatin for carvacrol is limited. The resulting nanofibers were beadless and droplet-free, exhibiting a uniform and smooth surface morphology. The fiber diameters ranged from the nanometer to the sub-micrometer scale, and the fibers were interlaced to form a three-dimensional porous network characterized by high porosity and a high specific surface area.

The practical value of CS-based NFs lies primarily in their ability to maintain EO activity at food surfaces over extended storage. They are therefore particularly suitable for fresh produce, berries, and other solid foods whose deterioration begins mainly at exposed interfaces. This suitability derives from the combination of high specific surface area, close interfacial contact, and fibrous diffusion resistance, which helps sustain localized antimicrobial and antioxidant protection while reducing premature EO dissipation. However, the same features may also limit rapid EO transfer in highly hydrated systems and make performance sensitive to moisture uptake, fiber uniformity, and fabrication reproducibility. In practice, NFs are most useful when sustained surface bioactivity is more important than rapid overall release [30].

### 3.2. Nanogels

CS-based NGs represent a gel-network approach to EO delivery in food preservation systems. Their main value lies in the presence of three-dimensional crosslinked domains that can confine active compounds, reduce premature losses, and regulate diffusion in hydrated environments. Unlike fibrous or particulate carriers, NGs are more closely associated with retention-oriented delivery and are therefore particularly useful when prolonged activity or environmentally responsive release is desired [6].

The retention behavior of CS-based NGs depends largely on polymer-active interactions and the internal organization created by crosslinking. Salah et al. reported an encapsulation efficiency of 83.14 ± 3.34% for cinnamaldehyde in CS gels [32]. Similarly, Hosseini et al. encapsulated Zataria multiflora EO in CS-caffeic acid nanogels, with an encapsulation efficiency of 82.69 ± 3.12% and an average particle size of 421.6 nm. Moreover, during a 60-day storage period, this nanogel system reduced pH fluctuations in cheese and maintained coliform counts within acceptable limits [33].

Crosslinking chemistry further determines whether gel-based CS systems function mainly as passive retention matrices or as responsive release platforms. Cheng et al. developed carboxymethyl CS/sodium alginate/carvacrol hydrogels using citric acid as a crosslinker, showing an encapsulation efficiency of 88.30 ± 1.46% [34]. The release of carvacrol from the hydrogel followed Fickian diffusion, with carvacrol being transferred from the interior to the exterior through diffusion and swelling upon water absorption. In addition, dissolution of the hydrogel also contributed to this process, releasing the carvacrol encapsulated in carboxymethyl CS.

CS-based NGs are therefore most suitable for hydrated food systems, including dairy products, acidic foods, and other matrices in which preservation benefits from prolonged activity while also requiring release behavior that varies with local conditions.

### 3.3. Nanoparticles

CS-based NPs act as discrete carriers for EO encapsulation in food preservation systems. Unlike fibrous networks, which mainly support surface-associated activity, NPs localize EOs within individual particles and allow more direct control over retention, interfacial contact, and diffusion-driven release. Their small size and high surface-to-volume ratio also facilitate rapid interaction with microbial or lipid interfaces, making them useful when efficient local delivery is required [27].

The performance of CS-based NPs is shaped not only by particle size, but also by polymer-related parameters and internal particle organization. Tea tree EO-loaded CS NPs had particle size of 340.07 ± 22.45 nm and encapsulation efficiency of 74.15–80.94%, and extended the shelf life of mini cucumbers by 9 days [35]. Curry leaf EO was loaded into CS NPs to prepare nanocapsules (encapsulated) and nanospheres (adsorbed), with average particle sizes of 547.9 ± 8.8 nm and 640.9 ± 18.1 nm, and EE of 66.91% and 61.39%, respectively [36]. These studies indicate that EO release from nanoparticles follows Fickian diffusion behavior. EO attached to the surface or embedded near the surface layer of the nanoparticles can diffuse rapidly into the medium, whereas EO in the CS core takes longer to diffuse through the polymer matrix. The CS matrix can absorb water and swell, allowing the EO to diffuse slowly from the pores.

Encapsulation of EOs in CS NPs enhances their stability and activity, thereby improving preservation performance. Thyme and oregano EOs were separately loaded into CS NPs, and nanoencapsulation improved their antioxidant and antimicrobial efficacy, as evidenced by reduced lipid and protein oxidation, enhanced radical scavenging activity, and better color and texture retention in meat emulsions [37].

These features make CS-based NPs particularly relevant for heterogeneous food systems, such as fresh produce and meat emulsions, where preservation depends not only on how much EO is present, but also on where and how it is delivered [35,36]. Their main advantage lies in discrete compartmentalization, high interfacial accessibility, and tunable release through particle size, polymer characteristics, and internal organization. These same features, however, also introduce complexity: smaller particles do not necessarily improve preservation if colloidal stability declines, and stronger encapsulation may depend on how effectively localized delivery, interfacial action, and release kinetics can be matched to the food microenvironment.

### 3.4. Nanoemulsions

CS-based NEs provide a droplet-mediated route for EO delivery in food preservation systems. Their main advantage lies in the formation of nanoscale oil droplets, which improve EO dispersion in aqueous media and increase interfacial contact with food surfaces or film matrices.

Reducing the particle size of incorporated EOs to the nanoscale through emulsification significantly increases the specific surface area of oil droplets. This results in improved stability as they become more uniformly distributed within the film-forming solution, thereby enhancing the utilization efficiency of EOs. CS films incorporating clove EO nanoemulsions showed a droplet size of 45.28 nm [38]. Eugenol-loaded CS nanoemulsions (144.3 ± 5.62 nm) better preserved postharvest guava quality than CS alone, maintaining firmness, soluble solids, titratable acidity, and total phenolics [39]. CS loaded with Eryngium campestre EO had an average droplet diameter of 75 nm and an EE of 81% [40]. This formulation significantly improved antioxidant performance, reduced lipid oxidation, and maintained the texture, sensory properties, and moisture content of ostrich meat over prolonged storage. Lavender EO-loaded curdlan/CS film, with a droplet size of 152.3 nm [41], significantly extended the shelf life of white mushrooms and straw mushrooms, reaching 12 days and 4 days, respectively. CS-based oregano EO NE coatings applied to apricots suppressed Alternaria alternata, significantly mitigating postharvest weight loss, retarding fruit softening, delaying the reduction in soluble solids content, and reducing both decay rate and disease index throughout storage [42].

A comparable matrix-level advantage was observed in ultrasound-assisted pullulan-based bioactive films containing EO-loaded NEs, in which improved organization of the active phase within the film contributed to more effective strawberry preservation during storage [4]. In this context, the main significance of NEs lies in improving EO distribution, retention, and functional persistence within matrix-based preservation systems rather than merely altering film properties.

In contrast to solid particulate carriers, NEs are particularly suitable for applications that depend on broad surface coverage and relatively uniform distribution of hydrophobic actives. This makes them especially relevant in edible coatings, postharvest treatments, and other preservation systems dominated by surface-related processes [43]. However, NEs are kinetically rather than thermodynamically stable, so their performance remains highly dependent on droplet stability, surfactant composition, and storage conditions.

### 3.5. Pickering Emulsions

CS-based PEs provide an interface-stabilized route for EO delivery in food preservation systems. Their defining feature is the adsorption of solid or colloidal particles at the oil-water interface, which creates a more robust interfacial layer than that of conventional surfactant-stabilized emulsions.

Shen et al. [44] developed Zanthoxylum bungeanum EO PEs stabilized by potato protein-CS NPs. The PEs had a droplet size of 280 nm and an FF of 92.91 ± 0.89%. Furthermore, the coating effectively prevented decay of citrus fruits stored at 25 °C caused by *Penicillium contamination*. In oregano EO PEs stabilized by ternary particles composed of zein-quercetin and quaternized CS to stabilize oregano EO and form Pickering nanoemulsions, with a droplet size of 900 nm and an oregano EO retention rate of up to 83.59% [45], Mottaki et al. prepared clove EO-loaded PEs stabilized by CS NPs, which exhibited a droplet size of 180.72 nm and an encapsulation efficiency of 98.85% [3]. In orange preservation tests, the inhibition effect of the PE showed no significant difference from that of the commercial fungicide Xedamix. These studies indicate that the dense capsule layer formed by NPs on the EOs droplet surface increases the resistance to EOs release, effectively prevents volatilization, and improves the storage stability of PEs [46,47].

CS-based PEs are particularly attractive for perishable fruits, vegetables, and processed meat products that require active droplets or coatings to remain effective over prolonged storage. Their suitability arises from particle-stabilized interfaces that suppress coalescence, reduce EO volatilization and oxidation, and maintain release over time more effectively than conventional surfactant-stabilized emulsions. However, this greater persistence is not cost-free. Stronger particle adsorption and more elaborate interfacial architectures may reduce release flexibility and increase difficulties in reproducibility, scale-up, and industrial standardization.

### 3.6. Nanocomposites

The incorporation of nanomaterials such as metals and metal oxides into film-forming CS matrices serves not only to improve structural integrity but also to influence the local distribution, retention, and release behavior of encapsulated EOs [48]. A PE stabilized by CS-TiO_2_ NPs encapsulating perilla EOs was prepared, with D_4_,_3_ and D_3_,_2_ values of 2.87 nm and 1.98 nm, respectively [49]. CS/PVA bionanocomposite films incorporating trans-cinnamaldehyde and TiO_2_ NPs formed denser, more continuous microstructures and extended mushroom shelf life from 6 to 10 days under refrigeration [50]. Nano-TiO_2_/daisy EO in CS composite films improved mechanical, antibacterial, and antioxidant performance, extending kiwiberry shelf life to 10 days [51]. These studies indicate that, in NCs, preservation is shaped not simply by EO release, but by the way nanofillers reorganize the matrix and thereby influence EO retention and local availability.

CS-based NCs are especially suitable for packaging systems in which active preservation must be achieved together with improved material performance, particularly when simultaneous control of EO delivery and matrix reinforcement is required. The drawback is that these same interactions can reduce release flexibility, and the incorporation of metal or metal oxide nanofillers raises additional concerns regarding dispersion homogeneity, migration, food-contact safety, and regulatory acceptance.

Table 1 summarizes the representative structural platforms for EO nano-delivery in CS-based active food packaging systems.

## 4. Effects of EO Nano-Delivery on the Physicochemical Properties of CS-Based Packaging

The incorporation of EOs into CS not only enhances its inhibitory activity against bacteria and its antioxidant properties, but also causes the formation and reorganization of the film’s network structure. CS molecules are rich in amino and hydroxyl groups, which can form electrostatic, hydrogen, and hydrophobic interactions with active components in EOs, such as alcohols, phenols, and aldehydes. This allows EOs to be stably loaded within CS. However, these interactions governing encapsulation are generally complex and are significantly influenced by the properties of CS, the composition of the EOs, and environmental factors. Consequently, the physicochemical properties of the CS-EO system are complex and diverse [52,53].

The incorporation of EOs into CS-based films primarily alters the film’s microstructure. This, in turn, influences key physicochemical properties, including microstructure, thickness, optical behavior, water-related properties, gas barrier performance, mechanical properties, and thermal stability. Generally, the uniform dispersion of EOs and a stable network structure contribute to enhanced mechanical properties and barrier performance. However, excessively high EO loading can lead to poor compatibility or increased agglomeration. This promotes the formation of voids, cavities, phase separation, and discontinuities in the film structure, thereby reducing the originally favorable physicochemical properties [52,54]. Therefore, it is essential to optimize the CS-EOs system to improve the physicochemical properties and freshness-preserving capabilities of the packaging material [41].

**Figure 1:**
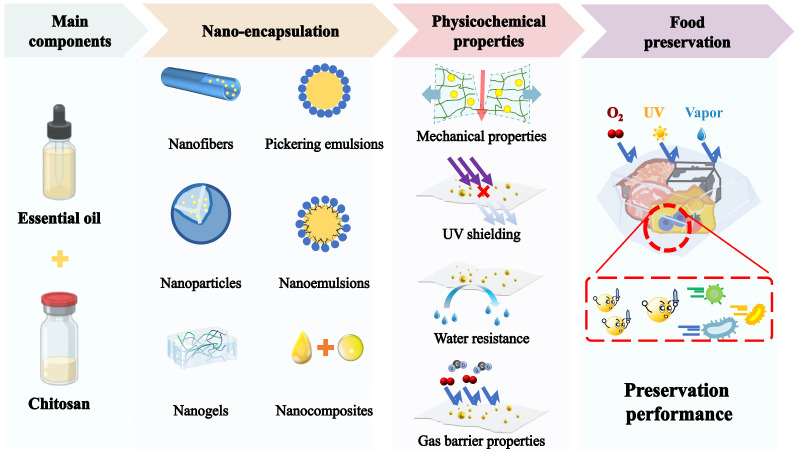
Schematic overview of the main components, nano-delivery platforms, physicochemical properties, and food preservation performance of CS-EO-based active packaging systems.

As summarized in Figure 1, CS and EOs can form six types of nanomaterials through different nanoencapsulation methods. The complex interactions between them influence the physicochemical properties and food preservation performance.

### 4.1. Microstructure

Among the physicochemical parameters discussed in this chapter, microstructure provides the most immediate structural evidence of how EO nano-delivery modifies the CS film network. Pure CS films usually exhibit relatively smooth surfaces and compact internal structures because strong intermolecular hydrogen bonding favors close chain packing. Once EOs are incorporated, however, a dispersed hydrophobic phase is introduced into the hydrophilic CS matrix, disrupting the original packing state and altering both surface and cross-sectional morphology. The resulting microstructural changes therefore reflect not a simple increase in roughness, but a broader process of network disturbance and reorganization following introduction of the oil phase [53].

Available studies show a broadly consistent trend: as EO loading increases, film structure tends to evolve from a relatively continuous state toward a more heterogeneous one. At low incorporation levels, overall continuity is often retained, although small pores, fine cavities, or slight irregularities may already appear on the surface or within the cross-section. For example, in the CS/TiO_2_/daisy EOs films, the incorporation of 0.5% DEO reduced surface cracks and particle precipitation. However, the presence of fine internal cavities was still observed, indicating that matrix reorganization had already commenced before continuity was seriously disrupted [51]. Likewise, in perilla EO/grape seed extract-CS/gelatin gel films, the optimized PE level was 6.91 μL/mL; under this condition, the film surface remained smooth, whereas the cross-section already showed a few pores, indicating that internal rearrangement may precede visible surface deterioration [55].

As the dispersed phase increases further, these early pores, cavities, and surface irregularities are more likely to develop into clear structural discontinuities. In fish skin collagen/CS films containing cinnamon EO, observations across the 0.1–0.4% CEO range showed that 0.2% produced the smoothest and most uniform morphology, whereas 0.3–0.4% led to grains, dense concavities, and rougher surfaces, indicating that structurally favorable organization occurred only within a limited addition range [56]. Similarly, in zein/CS films containing cinnamon EO-loaded PE, increasing the emulsion concentration from 0 to 1% improved surface uniformity and cross-sectional compactness, whereas 1.5% caused visible particles, cracks, and larger internal cavities [57].

Within this loading-dependent pattern, EO type and composition mainly influence how structural disruption is expressed rather than altering the overall direction of change. For example, in alginate-CS films, oregano and thyme EOs did not generate fundamentally different microstructural types at the same loading level; instead, increasing EO concentration remained the main factor associated with greater surface heterogeneity and more spongiform cross-sections [58]. By contrast, in a study where four EO NEs were introduced into the same CS system, all films showed good dispersion and compatibility at 5%, whereas cinnamon EO NE aggregated when the loading increased to 10% and 15% [52]. In addition, Citrus sinensis EO in carboxymethyl CS/peach gum films produced more obvious bulges, pits, and porous sponge-like cross-sections [59]. Taken together, these results suggest that loading largely determines the direction of microstructural evolution, whereas EO identity modulates the way such structural changes are manifested in specific systems. These differences are likely to influence subsequent thickness, moisture sensitivity, barrier behavior, and mechanical integrity [52,53].

### 4.2. Thickness

Thickness is one of the most direct macroscopic manifestations of the microstructural rearrangements described above. In EO-containing CS-based films, thickness reflects not only film geometry, but also changes in matrix packing, free volume, and drying shrinkage after introduction of the dispersed phase. EO-induced thickness variation is therefore better interpreted as the outcome of structural reorganization during film formation than as a simple increase or decrease in film size.

In many systems, EO incorporation leads to thicker films. This is commonly associated with an increase in the effective volume of the dispersed phase and reduced shrinkage during drying. For example, in zein/CS films containing cinnamon EO-loaded PE, film thickness increased from 0.073 mm to 0.091 mm as the emulsion concentration rose from 0 to 1.5%, corresponding to an increase of about 25%, indicating that EO-loaded dispersed phases can directly promote the formation of thicker film structures [57].

Thickness increase, however, is not the only possible outcome. In some systems, EO incorporation causes little or no significant change in thickness, even though other structural or functional characteristics are altered. For instance, in alginate-CS films containing oregano or thyme EO, thickness remained within 37.7–38.2 μm, with no significant differences across EO contents. This suggests that when the dispersed phase mainly fills pre-existing free volume or induces only limited rearrangement, macroscopic thickness may remain essentially unchanged [58].

In other cases, finer and more homogeneous droplet dispersion may promote tighter chain rearrangement during drying and thereby reduce film thickness. In PLA/CS bilayer films containing complex EO-loaded PE, the thickness ranged from 102.7 to 132.3 μm, and EO incorporation actually decreased thickness, with the highest EO treatment producing the thinnest film; this behavior was attributed to lower interfacial tension and more compact packing [53].

The effect of EOs on film thickness, therefore, reflects the balance between volume expansion and structural densification after introduction of the dispersed phase. When oil droplets or EO-loaded particles increase the effective volume and reduce drying shrinkage, the films tend to become thicker. When the dispersed phase mainly fills pre-existing voids, thickness may remain largely unchanged. By contrast, when dispersion is finer and drying-induced rearrangement becomes more compact, the thickness may decrease. This macroscopic response also provides a useful structural clue to subsequent changes in barrier properties and mechanical integrity [53].

### 4.3. Optical Properties

Optical properties of CS-based films are often noticeably altered after EO incorporation. These changes arise primarily from two factors: (1) the intrinsic color and light-absorbing characteristics of EO components, and (2) enhanced light scattering caused by dispersed droplets, pores, and internal inhomogeneities [53].

In many studies, the addition of EOs decreases CS-based film lightness while increasing its yellowness or brownness. For example, when a rosemary EOs nanoemulsion was added to CS-based films, the brightness decreased when the nanoemulsion content reached 1% or higher. Meanwhile, yellowness and total color difference began to increase at a 0.5% content, and the whiteness index also decreased as the EO content increased [60]. Similarly, in CS films containing clove EO NEs, increasing NE concentration from 0 to 1.0% continuously reduced L* while increasing b* and ΔE, indicating a progressive shift in overall film appearance [38]. In carboxymethyl CS/peach gum films loaded with 1.0% sweet orange EO, the a and b values of the films increased from 1.45 ± 0.17 and 6.89 ± 0.22 to 2.49 ± 0.18 and 11.3 ± 0.41, respectively, indicating that the films had a distinct yellowish tint [59].

Transparency and light transmittance are also commonly reduced after EO incorporation because most active components in EOs absorb ultraviolet light. The opacity of the carboxymethyl CS/gum arabic film loaded with sweet orange EO reached 12.53, which is 4.1 times that of the carboxymethyl CS/gum arabic film alone. Furthermore, the UV transmittance of this film in the 320–400 nm range was less than 15% [59]. In CS films loaded with daisy EO, the UV transmittance below 280 nm is similar to that of pure CS films, but the absorption rate increases slightly above 302 nm, which may be attributed to the UV-absorbing properties of the compounds in daisy EO [51]. Xue et al. demonstrated that simultaneously incorporating EOs and nano-titanium dioxide into CS films can significantly reduce UV transmittance, with transmittance levels below 10% or even 0% across the entire UV spectrum [55].

Ultraviolet radiation triggers photochemical reactions that degrade food quality, with particularly significant effects on light-sensitive foods. The film exhibits strong UV-blocking capabilities, indicating its potential application in food preservation by extending shelf life through the prevention of UV-induced degradation [38,60].

### 4.4. Water Solubility and Moisture Content

Because CS is intrinsically hydrophilic, its films commonly exhibit relatively high moisture content, considerable water solubility, and pronounced moisture sensitivity. EO incorporation can often alleviate these limitations, although the extent of improvement still depends on dispersion state and loading level [53].

In most systems, EOs reduce water sensitivity through two main pathways. First, the hydrophobic oil phase decreases direct contact between water and the polar groups of the polymer network. Second, when the dispersed phase is incorporated in a relatively well-organized manner, it can also restrict water diffusion through the film. For instance, in buckwheat starch/CS coatings containing curry leaf EO, MC decreased from 13.35% to 10.01%, and WS decreased from 31.77% to 26.25%, indicating a clear reduction in water affinity after EO incorporation [61]. Likewise, in clove EO NE-loaded CS films, the 0.6% CEO treatment showed a higher water contact angle and lower water solubility, suggesting that a moderate NE level can simultaneously improve hydrophobicity and water resistance [38]. In CS nanofibre films loaded with oregano EOs emulsion, the mass loss of the swollen films decreased from 40% to 23% as the concentration increased, indicating improved water stability [62].

This improvement, however, does not increase indefinitely with EO content. In pH-responsive zein/CS composite films containing cinnamon EO-loaded PE, MC, and WS initially decreased and reached 16.18% and 10.92%, respectively, at 1% emulsion loading, but both increased again at 1.5% emulsion loading, indicating that excessive EO can weaken water resistance by introducing structural defects [57].

The effect of EOs on WS and MC is therefore better understood as the combined consequence of hydrophobic shielding and structural integrity. Moderate EO dispersion typically reduces water uptake and solubility, whereas excessive loading or poor dispersion may reopen diffusion pathways via newly formed pores, cavities, or local discontinuities [38,57,61]. In this sense, improvements in water-related properties are not merely compositional effects of adding a hydrophobic phase, but structural outcomes that depend on whether EO incorporation stabilizes or disrupts the film network.

### 4.5. Gas Barrier Properties

The effect of EOs on gas barrier properties is governed less by the mere presence of an oil phase than by the way that phase modifies moisture sorption and transport pathways within the film. In CS-based films, water vapor and gas permeation are usually reduced when the dispersed phase is well incorporated, decreasing free volume and making the diffusion path more tortuous. Once droplet aggregation, phase separation, or major defects appear, this barrier advantage is typically weakened [53].

This pattern is evident in several systems. The WVP of zein/CS film with black wolfberry anthocyanins was 0.33 g·mm/kPa·h·m^2^. Adding 0.5–1.5% cinnamon EOs-loaded PEs first increased, then decreased the WVP. At 1% PEs, the WVP was 0.21 g·mm/kPa·h·m^2^, showing the best water vapor barrier performance [57]. This result is consistent with the findings reported by Xue et al. [55], who observed similar changes in WVP when perilla EOs were added to CS/gelatin edible gels. At 10 μL/mL, the WVP exhibited its lowest, measuring only 1.36 × 10^−5^ g·cm/kPa·h·cm^2^. Rui et al. [38] found that for CS films loaded with 0.2–1.0% (*v*/*v*) clove EOs-loaded nanoemulsions, the trend in OP was similar to that of WVP. The lowest values were both recorded at 0.6% (*v*/*v*), with a WVP of 0.56 × 10^−6^ g/Pa·h·m and an OP of 2.362 × 10^−3^ g/m·s. These results indicate that hydrophobic EOs can fill the micropores or cracks and limit the diffusion of water and O_2_ molecules in the film, thereby enhancing its barrier properties. However, an excess of EOs can lead to phase separation and interfere with the cross-linking between the film matrix components. This may disrupt the film structure and reduce its gas barrier properties.

The gelatin/CS gel film containing 10 μL/mL perilla EOs helped to maintain the water content of the grass carp, which declined slowly from 76% to 55% [55]. This is because the gel film blocks oxygen and reduces moisture evaporation, thereby mitigating water loss from the fish.

### 4.6. Mechanical Properties

EO incorporation redistributes the balance between structural cohesion and chain mobility, and its effect on the mechanical properties of CS-based films is therefore not unidirectional. In some systems, a moderately dispersed EO phase improves network coordination and increases strength. In others, the oil phase behaves more like a plasticizer, making the film more flexible but less resistant to tensile stress. Once loading becomes excessive and induces aggregation or phase separation, mechanical integrity usually deteriorates [53].

In systems that follow a reinforcement-dominated path, a moderate dispersed phase can improve matrix coordination. For example, in pH-responsive zein/CS composite films, the addition of cinnamon EO-loaded P increased TS from 13.40 to 25.49 MPa, whereas EAB decreased from 36.38% to 16.01%, indicating reinforcement at the expense of flexibility. When emulsion loading increased further to 1.5%, both TS and EAB declined to 19.51 MPa and 9.39%, showing that excessive dispersed phase ultimately undermined the reinforcement effect [57].

In systems that follow a more pronounced plasticization path, EOs mainly weaken matrix cohesion. In CS films containing clove EO-loaded microemulsions, TS decreased from 9.89 to 3.50 MPa, while EAB increased to 45.06% at 0.6% and then dropped to 30.51% at higher loading, indicating that low-to-moderate microemulsion levels improved deformability, whereas higher levels weakened overall mechanical integrity because of structural discontinuity [63]. Likewise, in clove EOs-loaded NE CS films, higher CEON concentrations reduced TS, while the 0.6% CEO film showed a higher EAB, suggesting that a moderate NE level can improve flexibility to some extent [38].

The effect of EOs on mechanical performance is therefore better interpreted as a dynamic balance among reinforcement, plasticization, and structural disruption. The decisive factor is not simply whether EO is present, but how it is dispersed within the matrix and whether that state improves network coordination or weakens structural continuity. Mechanical changes should therefore be read together with the preceding microstructural and thickness responses rather than as isolated property shifts.

### 4.7. Thermal Stability

Thermal stability reflects the ability of a film material to resist water loss, chain scission, and structural degradation during heating. Compared with the CS film, the CS nanofiber film loaded with oregano EO emulsion showed lower mass loss and a higher maximum thermal degradation temperature (Tmax) in TGA and DTG [62]. Jannah et al. incorporated 20% (*v*/*v*) NEs of cinnamon EOs into sodium alginate/CS bilayer film. TGA results showed that adding the NEs increased the Tmax of the first and second transition phases from 235 °C and 282 °C to 236 °C and 288 °C, respectively. Additionally, a new transition peak appeared at 386 °C [64]. Gao et al. incorporated NEs of four EOs (cinnamon, clove, thyme, and tea tree) at 5% (*v*/*v*) into CS films. TGA showed that all NEs reduced the weight loss of the films [52]. These studies indicate that the interactions between EOs and CS enable the successful loading of EOs onto CS-based films and enhance their thermal stability. Furthermore, TGA/DTG analysis was performed on CS films loaded with 5–15% (*v*/*v*) cinnamon EO-loaded NEs. The results indicated that the lowest weight loss occurred at 5% loading, with the smallest peak area and lowest decomposition rate at temperatures between 30 and 140 °C [52]. This may be due to the aggregation of CEO emulsion droplets, which disrupts the matrix and leads to weight loss. Notably, the effect of EOs on thermal stability follows the same trend as that on other physicochemical properties discussed above.

The main structure-property relationships of EOs nano-delivery in CS-based films are summarized in Table 2.

## 5. Smart Packaging Functions of CS-EO Nano-Enabled Systems

CS-EO nano-enabled systems are being increasingly investigated for smart packaging, which transforms passive preservation into an active and adaptive process. From a functional perspective, CS-EO smart packaging can be divided into two categories: freshness-indicating packaging and stimulus-responsive controlled-release packaging.

Freshness-indicating packaging uses pH-sensitive or redox-sensitive indicators that change color to show the degree of spoilage, providing an instant, non-invasive indicator of food quality [65,66]. This makes it particularly useful in situations where consumers would otherwise struggle to assess a product’s freshness. In freshness-indicating packaging, EOs do not directly cause color changes, but they are key auxiliary components of this smart packaging. Thanks to their exceptional antimicrobial and antioxidant properties, EOs enhance indicator stability and ensure this smart packaging maintains clear, accurate color reactions under various storage conditions.

Stimulus-responsive controlled-release packaging responds to specific triggers (pH, microbial growth, or enzyme activity) and environmental conditions (temperature, humidity, or moisture), ensuring that active ingredients are released only when necessary. This maximizes their effectiveness and enables them to adapt to the changing environmental conditions encountered during food storage and transportation [66,67].

**Figure 2:**
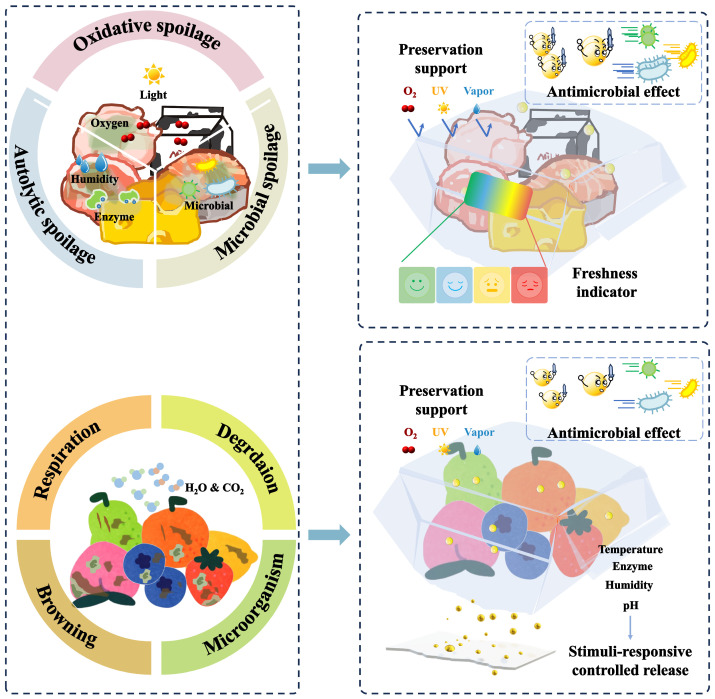
Schematic representation of freshness-indicating and stimuli-responsive packaging functions of CS-EO nano-enabled systems for food preservation.

As illustrated in Figure 2, these two functional routes together define the principal smart-packaging applications of CS-EO nano-enabled systems in food preservation.

### 5.1. Freshness-Indicating Packaging

EOs can enhance the compatibility of natural pigments with CS and other matrices. This enables natural pigments to exhibit excellent color stability and respond to changes in pH, acetic acid, and ammonia. Furthermore, the exceptional antimicrobial and antioxidant properties of EOs inhibit microbial growth and the oxidation of proteins and lipids during food spoilage. This results in more pronounced color changes in CS-based freshness-indicating packaging, with smoother color transitions, and clearly distinguishable intermediate shades. However, adding EOs is not always beneficial, as higher concentrations may compromise the sensitivity of natural pigments, thereby reducing their effectiveness as pH indicators in smart food packaging.

#### 5.1.1. Single-Phase Integrated Systems with Coexisting Indicators and Active Components

The defining feature of single-phase integrated systems is that the indicator, CS matrix, and EO-related components coexist within the same film phase, allowing one material to provide both visual indication and active preservation. Lv et al. [68] reported that incorporating cinnamaldehyde EO into alizarin/CS/agar film resulted in excellent hydrophobicity, color stability and responsiveness. Compared with films devoid of EO, these films exhibited greater color alteration and distinction across various pH conditions. Specifically, yellow (acidic), brown (neutral), purplish-red (weakly alkaline), and deep purple (strongly alkaline). Furthermore, the film exhibits distinct colorimetric responses to acetic acid (from purplish-red to reddish-brown to orange, ΔE > 5) and to ammonia (from purplish-red to deep purple). As fresh-cut papaya and shiitake mushrooms spoil, labels show distinct color changes (ΔE > 15), with purple turning to purplish-red and to brown, respectively. Similarly, Talebi et al. [69] observed enhanced pH-responsive color changes and ammonia gas sensitivity with the addition of ginger EO-loaded PEs to aronia anthocyan/CS/kappa-carrageenan films. This molecular arrangement alters the film microstructure, which reduces the interactions between the pigment and the surrounding aqueous environment, thereby slowing its pH-driven structural transformation. Consequently, a more gradual color transition with clearly distinguishable intermediate hues is observed.

When EOs and natural pigments are present in the same phase, high concentrations may directly impair the sensitivity of the natural pigments. This would reduce their effectiveness as pH indicators in smart food packaging. Zhu et al. [57] and Lim et al. [70] found that the microstructure of the film changed when the concentration of cinnamon EO-loaded PEs reached 1.5%. This weakened the interaction between anthocyanins and the surrounding pH, thereby inhibiting their pH responsiveness. Similarly, Chen et al. prepared cornstarch films containing tangerine peel EO-loaded PEs and purple corn anthocyanins displayed similar trends [71]. Therefore, it is necessary to optimize the formulation and compatibility of the components in this single-phase system.

#### 5.1.2. Layered or Spatially Separated Systems for Sensing and Preservation

The freshness-indicating layer and the EO-loaded preservative layer are separated using methods such as layering or encapsulation. This reduces the adverse effects of excess EOs on anthocyanins, thereby improving the accuracy of freshness monitoring in packaged foods.

Wu et al. prepared a multifunctional bilayer film via layer-by-layer casting, consisting of cinnamaldehyde NE/CS antimicrobial layer and alizarin/sodium alginate indicator layer [72]. The film exhibits a sensitive response to pH changes, gradually changing from yellow to red and eventually to purple as the pH rises from 2 to 11. Notably, the film undergoes a reversible color change in 2 min and remains sensitive after several cycles. In experiments on shrimp preservation, the film’s color changed from brown to purple as spoilage progressed (ΔE > 5), extending the shelf life of the shrimp to 46 h. Consistent with these results, Huang et al. [73] and Liu et al. [74] prepared multifunctional bilayer films. Both types of films were sensitive to ammonia, and their color changes showed a linear correlation with TVB-N in fish meat and a positive correlation with TVB-N and total bacterial count in pork, respectively. Zhang et al. [75] prepared antimicrobial indicator cards by coating both sides of filter paper with an indicator layer loaded with red cabbage anthocyanins and a layer of illite nanotubes loaded with thyme EO, respectively. These indicator cards show excellent pH sensitivity and ammonia responsiveness, with color changes, and can extend the shelf life of fish by 1 to 2 days.

Compared with single-phase integrated systems, the incorporation of a protective layer can improve the stability of natural pigments. However, multilayer composite film technology involves complex preparation processes, which raises concerns about performance consistency in large-scale production.

#### 5.1.3. Multi-Signal or Multimodal Integrated Systems

When a single-color signal is insufficient for reliable judgment under complex storage conditions, multi-signal or multimodal systems improve interpretive reliability through complementary outputs. Recent reviews have identified colorimetric sensor arrays as an important direction in freshness-indicating packaging because, compared with single-component films, multi-point systems often provide higher sensitivity and broader analyte coverage.

Peighambardoust et al. developed a dual-sensor label that combines bromocresol purple and methyl red and used it together with a CS coating containing yarrow EO for monitoring chicken filet freshness and extending shelf life [76]. The dual-sensor label distinguished fresh, semi-fresh, and spoiled stages more clearly than a single readout. Lin et al. further demonstrated multimodal integration using a double-layer system in which the outer layer provided a colorimetric response, whereas the inner layer combined a fluorescence response with cinnamaldehyde-related functional components for pork freshness monitoring and preservation [77]. In that study, pork shelf life was extended by at least 2 days, and pork was considered inedible when ΔE ≥ 12 or fluorescence intensity ≤ 21.54. Thus, the advantage of multimodal design lies not simply in adding signals, but in improving interpretive robustness under variable spoilage conditions.

At the same time, signal integration, module coordination, and result interpretation become more complex. The practical value of multimodal systems therefore depends on whether the added information can improve decision-making without undermining usability.

Representative freshness-indicating CS-EO systems are summarized in Table 3.

### 5.2. Stimuli-Responsive Controlled-Release Packaging

Traditional active packaging extends the shelf life of food by releasing antimicrobial agents and antioxidants, but the release of these active ingredients lacks selectivity. Researchers have recently explored responsive packaging that can detect spoilage signals (e.g., pH changes, microbial proliferation, or increased enzyme activity) or environmental fluctuations (e.g., changes in temperature, humidity, or moisture content). These systems enable more precise and efficient food preservation by releasing active substances in a targeted manner under specific trigger conditions.

CS has a highly modifiable structure, with each glucosamine unit bearing three reactive sites, including an amino group at C_2_ and hydroxyl groups at C_3_ and C_6_. This allows for chemical modifications that introduce responsiveness to pH, temperature, enzymes, or reactive oxygen species, thereby providing a structural basis for multi-responsive smart packaging materials.

In these systems, EOs function both as core active agents and as modulators of film responsiveness by adjusting the compactness and hydrophobicity of the CS film.

#### 5.2.1. pH-Responsive Release

Among available triggers, pH variation is one of the most widely studied because it is closely linked to the spoilage microenvironment of foods.

CS can form Schiff base bonds with other biopolymers, which are prone to cleavage or relaxation under acidic conditions, thereby promoting the release of EOs or EO-derived active compounds. CS and fucoidan form Schiff base imine bonds and encapsulate cinnamaldehyde, creating a film with pH-responsive and controlled-release properties [80]. The amount of cinnamaldehyde released increased as the pH decreased, and the antibacterial rate at pH 5 was correspondingly 2.3-fold higher than that at pH 7, indicating good pH-responsive antibacterial properties in mildly acidic environments. Owing to its excellent performance, this film significantly prolonged the shelf life of litchi fruits (>8 d). Wen et al. [81] and Zhang et al. [82] also drew similar conclusions and prepared two types of pH-sensitive antimicrobial films via Schiff-base imine bonding, using CS/cinnamaldehyde and carboxymethyl CS/cinnamaldehyde, respectively. Both films exhibited pH-responsive active release properties. The CS-cinnamaldehyde film extended the shelf life of chilled mutton to 4 days at 4 °C, while the carboxymethyl CS-cinnamaldehyde film doubled the shelf life of longan and citrus and effectively reduced moisture loss.

Li et al. prepared nanocapsules encapsulating lavender EO through electrostatic interactions between CS and sodium tripolyphosphate, as well as a Schiff base reaction between CS and glutaraldehyde [83]. Under alkaline conditions (pH 10), the release rate of EO increased significantly, confirming their pH-responsive properties. Similarly, calendula EO encapsulated in CS NPs also exhibited pH-responsive controlled-release properties. Li et al. [84] developed a pH-responsive CS/polyvinyl alcohol/polyvinylpyrrolidone hydrogel film loaded with clove EO, which demonstrated pH-controlled release of clove EO. This mechanism is attributed to the protonation of amino groups in CS under acidic conditions, which induces electrostatic repulsion, network swelling, and ultimately accelerates the release of clove EO. This film extended the shelf life of blueberries by at least 6 days.

Its effectiveness, however, still depends on the magnitude of pH fluctuations in the food matrix, the chosen response threshold, and the stability of dynamic bonds under complex storage conditions. The key issue is therefore not simply whether pH can trigger release, but whether trigger intensity and material sensitivity are properly matched.

#### 5.2.2. Humidity-Responsive Release

Unlike pH-responsive systems, humidity-responsive release depends mainly on moisture-induced physical reconfiguration of the packaging matrix. This makes humidity especially relevant for fruits, vegetables, and other high-moisture foods, where water-vapor accumulation is difficult to avoid. As relative humidity increases, the CS matrix absorbs water and swells, the intermolecular spacing expands, and diffusion pathways become more accessible, thereby facilitating EO migration and release.

A humidity-responsive hydrogel pad based on carboxymethyl CS/gelatin loaded with thyme EO demonstrated that humidity effectively regulated the release of thyme EO, with cumulative release rates of 10.01 ± 0.27%, 32.65 ± 2.10%, and 55.59 ± 0.77% at 30%, 60%, and 90% relative humidity, respectively. Moreover, this hydrogel pad kept straw-berries mold-free for 20 days, extending their shelf life by more than 15 days [85]. This can be attributed to the fact that under high humidity, water molecules induce swelling of the polymer network, increase matrix pore size, and reduce diffusion resistance, thereby promoting EO release. A humidity-responsive membrane prepared by encapsulating cinnamaldehyde-loaded halloysite nanotubes into CS/polyvinyl alcohol fibers extended strawberry shelf life by approximately 150% at 25 °C [86]. Similarly, an aerogel fabricated by incorporating cinnamaldehyde-loaded β-cyclodextrin into CS/dialdehyde nanocellulose showed significantly increased release when relative humidity was raised from 70% to 98%, and strawberries packaged with this aerogel remained in good condition after 5 days of storage at 22 ± 1 °C [87]. Unlike the release behavior of EOs directly added to the CS matrix, EOs loaded in halloysite nanotubes and β-cyclodextrin must first be released from these carriers before diffusing into the CS matrix. Consequently, no obvious initial burst release is observed in the release profiles. The limitation is specificity. Because humidity is broadly sourced and highly variable, release behavior may be affected not only by spoilage progression but also by hygroscopicity, environmental fluctuation, and food respiration intensity. Humidity-responsive systems are therefore practically relevant, but they are harder to regulate precisely.

#### 5.2.3. Temperature-Responsive Release

Whereas pH and humidity mainly reflect changes inside the package, temperature variation is more closely associated with disturbances along the food supply chain. Fluctuations during processing, transport, distribution, and household storage can alter the chain mobility and interfacial state of the CS matrix, thereby affecting EO diffusion and volatilization. The key value of temperature-responsive systems lies not in the simple observation that release accelerates at higher temperature, but in whether structural design can suppress premature EO loss under low-temperature storage while enabling rapid intervention during temperature abuse.

Ren et al. prepared a coating by encapsulating trans-2-hexenal in thermosensitive liposomes assembled from open-chain phospholipids, combined with CS-stearic acid nanoclusters. This coating enabled temperature-modulated release (10–40 °C), improving peach quality and extending shelf life to 10 days [88]. Li et al. synthesized CS-g-PNIPAm microcapsules encapsulating mugwort oil via ATRP, showing release rates of 79.2%, 92.2%, and 96.1% at 20, 40, and 60 °C after 48 h, respectively [89]. As temperature increases, molecular motion intensifies, van der Waals forces weaken, and hydrogen bonds between molecular chains are disrupted, leading to a rapid increase in EO release. These results show that incorporation of thermoresponsive segments can convert temperature variation into tunable EO release. Du et al. [90] prepared a composite film based on eugenol@porous microspheres@modified CS quaternary ammonium salt, which achieved a CO_2_/O_2_ selective permeability ratio of 5.58 at 25 °C while exhibiting temperature-responsive antimicrobial release. This film extended the postharvest shelf life of bananas and kiwifruit to 3 days and 9 days, respectively. Wang et al. [91] co-encapsulated cinnamaldehyde and linalool in a Pickering emulsion based on CS-ε-polylysine-sodium tripolyphosphate. The release rates of cinnamaldehyde and linalool steadily increased with rising temperature over 5 days, reaching a maximum of 66.6% at 40 °C.

Compared with other single-stimulus systems, temperature-responsive systems are particularly relevant to practical scenarios such as cold-chain instability and temperature abuse. However, their design is often complex and awkward, offering no cost or fabrication advantages, so they are not suitable for large-scale production outside of laboratories.

#### 5.2.4. Enzyme-Responsive Release

Compared with environmental triggers such as pH, humidity, and temperature, enzyme-responsive release is more closely tied to the biological basis of food spoilage. Its underlying strategy is to trigger EO release through the direct action of extracellular enzymes secreted by microorganisms on gatekeeping layers, responsive shells, or cleavable structural motifs. Because the triggering source is tightly coupled to microbial activity itself, such systems offer a higher degree of biological specificity.

Lin et al. embedded citric acid@hyaluronic acid/carboxymethyl CS NPs into silk fibroin-based nanofibers to prepare enzyme-responsive intelligent packaging. Hyaluronic acid and silk fibroin can be hydrolyzed by β-glucuronidase and serine protease secreted by *E. coli* O157:H7, respectively, triggering release. Consequently, the nanofibers exhibited significant inhibitory effects against *E. coli* O157:H7 and demonstrated dual-responsive smart controlled-release properties [92]. Gao et al. prepared an intelligent composite pad by embedding gelatin-CS microcapsules encapsulating a cinnamon-oregano EO blend into a PVA/konjac glucomannan (PK) matrix. The pad specifically triggers EO release upon gelatinase secretion by spoilage bacteria, enabling on-demand antimicrobial and antioxidant activities. In chilled pork preservation tests, the pad extended shelf life by at least 8 days at 4 °C [93]. Compared with proof-of-concept studies, this example more clearly shows that enzyme-triggered EO release can translate into practical preservation benefits.

Enzyme-responsive systems are particularly appealing because they can selectively suppress the main spoilage microorganisms and enhance preservation. Nevertheless, research in this area is still at an early stage, and future work should focus on system simplification, broader applicability, and improved performance robustness.

#### 5.2.5. Multi-Stimuli-Responsive Controlled-Release Systems

Real food spoilage rarely depends on a single variable. Acidification, enzyme secretion, humidity increase, and external disturbances often occur together. Multi-stimuli-responsive systems are therefore not merely combinations of several triggers; their purpose is to couple different signals so that release behavior more closely reflects the complexity of actual spoilage processes.

Du et al. developed a dual pH- and amylase-responsive antimicrobial nanofibrous membrane (thymol@PLA/CS oligosaccharide/dialdehyde starch). Either acid cleavage of Schiff-base bonds or amylase hydrolysis of dialdehyde starch opens the dialdehyde starch “gatekeeper”, enabling controlled thymol release. Rhizopus on melons secretes both acid and amylase, which act on these two responsive structures, achieving on-demand release and extending melon shelf life to 11 days [94]. Jiao et al. [95] prepared a cinnamaldehyde@PVA/CS-Cin film with humidity- and pH-responsive release. At 30% and 80% RH, cumulative cinnamaldehyde release over 60 h reached 5.09 and 18.46 μg/mg, respectively; at pH 4 and 7, 12 h release was 6.36 and 2.16 μg/mg, respectively. This dual-responsive film releases cinnamaldehyde under high humidity to inhibit microbes and rapidly releases more upon pH drop from mold growth, effectively extending strawberry shelf life. Their design challenge is correspondingly greater. Interactions among stimuli, trigger sequence, threshold setting, and differences among food systems all complicate optimization. Multi-stimuli responsiveness is therefore a promising direction, but its design remains complex and difficult.

Representative stimuli-responsive CS-EO systems are summarized in Table 4.

## 6. Challenges and Future Perspectives of CS-EO Nano-Enabled Packaging Systems

CS-EO nano-enabled smart packaging systems have attracted increasing attention as multifunctional platforms that combine active preservation with real-time quality monitoring. By integrating the film-forming and antimicrobial properties of CS with the bioactivity of EOs and the tunable functionality of nanostructured carriers, these systems can support both freshness indication and controlled release of active compounds [96,97,98]. Despite encouraging laboratory-scale results, the translation of these systems into practical commercial applications remains limited. The current challenge is no longer confined to material-level optimization, but lies in how to simultaneously address multiple issues, including global regulatory alignment, manufacturing scalability, and consumer acceptance. These aspects must be advanced synergistically to achieve successful industrial translation.

### 6.1. Safety Considerations

Safety is a prerequisite for the practical deployment of CS-EO nano-enabled smart packaging systems. Although CS is widely regarded as a biocompatible and biodegradable polysaccharide, and several EOs or their constituents are already used in food-related applications, the safety of the final nano-enabled packaging system cannot be inferred from its individual components alone [99]. System-level evaluation is required because nanostructuring can alter the retention, release, migration, and bioavailability of active substances in food-contact environments [5,25].

Source heterogeneity is a major concern. CS obtained from different raw materials may vary in purity, degree of deacetylation, molecular weight, and residual impurities, all of which affect both functionality and safety. Incomplete deproteinization of crustacean-derived CS may leave allergenic residues, whereas EOs can differ markedly in activity, sensory impact, and toxicological profile depending on botanical origin, composition, and dose [99,100,101]. Nanocarriers may further alter the partitioning of active compounds among the packaging matrix, headspace, and food, thereby changing actual consumer exposure during storage [5,98].

Future studies should adopt a safety-by-design framework. This requires rigorous raw-material characterization, control of residual impurities, rational EO loading, migration testing with relevant food simulants, and long-term exposure assessment under realistic storage conditions. Advanced chromatographic and mass-spectrometric methods will be essential for identifying and quantifying migrating compounds, while toxicological evaluation should focus on the final packaging system rather than on isolated components alone [102,103].

### 6.2. Regulatory Challenges

Regulatory approval remains a major obstacle to commercialization. Because CS-EO nano-enabled smart packaging systems are intended for direct food contact, both the structural matrix and the active compounds must comply with food-contact legislation. The challenge becomes greater when nanoscale structures are involved, since nanomaterials may show size-dependent physicochemical behavior, migration characteristics, and biological interactions that differ from those of conventional materials [104,105].

In the European Union, active and intelligent food-contact materials are governed by Regulation (EC) No. 450/2009, while nano-enabled materials increasingly require nano-specific characterization and risk assessment. More broadly, recent reviews emphasize that regulatory evaluation of nano-enabled packaging should consider not only chemical composition, but also particle-size distribution, migration potential, and exposure after gastrointestinal transformation or transfer from packaging to food [96,104,106].

Several issues remain unresolved. From a global perspective, the regulatory approaches toward nano-enabled food-contact materials vary significantly across regions. The European Union operates under a precautionary positive list system established by the Framework Regulation (EC) No. 1935/2004, which requires all substances, including nanomaterials, to undergo explicit safety assessment and authorization before they can be used. In contrast, the U.S. Food and Drug Administration employs a more flexible premarket notification system under 21 CFR, regulating these materials as food contact substances through its Food Contact Notification program. Meanwhile, while China’s GB 4806 series of standards establishes general safety requirements for food-contact materials, it currently lacks specific provisions and guidance tailored to nanomaterials. These divergent regulatory frameworks impose a substantial compliance burden on global product deployment, since a material authorized in one region may not necessarily gain approval in another. Therefore, harmonizing migration-testing methods and risk assessment standards for nanomaterials remains an urgent and unresolved challenge. Many current frameworks were developed for conventional food-contact materials and do not fully address the complexity of nano-enabled organic or hybrid systems. Migration-testing methods for nanomaterials also remain insufficiently harmonized across jurisdictions. In addition, CS-EO systems are multifunctional by nature, often combining a biopolymer matrix, nanostructured carriers, and active antimicrobial agents, which may place a single packaging material under multiple regulatory categories and complicate approval procedures [104,106].

Future work should therefore support regulatory harmonization through validated methods for nanomaterial migration analysis, standardized exposure scenarios, and case-specific toxicological testing strategies. Closer alignment between material development and regulatory science will be essential if CS-EO nano-enabled smart packaging systems are to move from laboratory studies to market approval. Otherwise, regulatory uncertainty may remain a practical barrier even when technical performance is satisfactory.

### 6.3. Stability of CS-EO Nano-Delivery Systems

The functional performance of CS-EO nano-enabled smart packaging systems depends strongly on the stability of the nano-delivery system itself. EOs are inherently volatile and prone to oxidation, which can reduce the concentration of active constituents and weaken antimicrobial efficacy during processing and storage [102,107]. Although encapsulation can improve retention and protect labile compounds, carriers may still undergo aggregation, phase separation, interfacial destabilization, or matrix degradation, ultimately causing premature release or uneven distribution of active agents [7,25].

Environmental stresses such as temperature fluctuations, humidity, oxygen, and light can further accelerate these processes. Stability assessment should therefore extend beyond initial encapsulation efficiency to include post-processing integrity, storage resistance, volatile retention, and preservation of antimicrobial activity under relevant application conditions. Strategies such as multilayer encapsulation, cyclodextrin inclusion complexes, interfacial reinforcement, and antioxidant co-stabilization may improve retention and release control, but their value must be verified after film formation and under actual food-storage conditions rather than only in freshly prepared dispersions [108,109,110]. Related evidence from dual-crosslinked CS gel-like systems containing flavonoid extract and cinnamaldehyde oil further suggests that network design can improve functional integration and help preserve antioxidant and antimicrobial performance, indicating that carrier stabilization and active retention need to be addressed together rather than separately [32].

### 6.4. Reliability and Comparability Across Studies

Beyond chemical and structural stability, the intelligent functions of CS-EO nano-enabled smart packaging systems must remain reliable during storage, transportation, and retail handling. Local variation in humidity, temperature, gas composition, pH, and food-derived metabolites can perturb the NC microenvironment and thereby alter both signal output in freshness indicators and release behavior in stimuli-responsive systems [65]. Good laboratory performance therefore does not necessarily translate into robust real-world functionality. Reliability should be treated as an independent translational requirement rather than as a secondary consequence of material stability alone.

#### 6.4.1. Reliability of CS-EO Freshness Indicator Systems

The reliability of CS-EO freshness indicator systems depends on maintaining a stable and interpretable sensing microenvironment around pigments or other responsive moieties. In real storage environments, water uptake, local pH shifts, volatile amines, carbon dioxide, and EO redistribution can alter polarity, moisture content, and acid-base balance within the matrix. These changes may shift color-transition thresholds, weaken signal intensity, or generate spatially heterogeneous responses, thereby compromising indication accuracy [111,112].

Current studies still rely heavily on controlled laboratory conditions and visible color change as the main proof of performance. Future work should place greater emphasis on calibration under realistic supply-chain conditions, quantitative evaluation of false-positive and false-negative responses, and improved control of pigment distribution, diffusion, and stabilization within the matrix. Promising approaches include self-healing or dynamically crosslinked CS-based networks, multifunctional or dual-sensor indicator labels, and digital image-assisted signal interpretation, all of which may improve signal reproducibility and reduce subjectivity under complex storage conditions [12,113].

#### 6.4.2. Reliability of CS-EO Stimuli-Responsive Release Systems

For CS-EO stimuli-responsive release systems, the key challenge is not simply to release EOs, but to release them at the right time, at an appropriate rate, and at the relevant location. Under practical storage conditions, fluctuations in humidity, temperature, pH, and headspace composition alter matrix swelling, polymer-chain mobility, and diffusion pathways, while interactions among EO molecules, nanocarriers, and the CS matrix affect retention and redistribution. These coupled effects may lead to burst release, delayed release, or spatially heterogeneous release, resulting in inconsistent antimicrobial protection [67].

To improve reliability, the release design should be linked more closely to the spoilage behavior of specific food systems. Future work should combine tunable crosslink density, multilayer or gradient architectures, and carrier functionalization with release-kinetics modeling and in situ monitoring. Such an integrated strategy would enable more predictive control over when and how active compounds are delivered, thereby improving preservation consistency under dynamic storage conditions [34,114].

### 6.5. Manufacturing Scalability and Economic Constraints

Industrial translation also depends on whether CS-EO nano-enabled smart packaging systems can be produced at scale with acceptable cost and reproducible quality. High-purity CS and food-grade EOs can be relatively expensive, while commonly used fabrication methods, such as nanoemulsification, electrospinning, and encapsulation-based assembly, are often batch-dependent, equipment-intensive, and sensitive to processing parameters. These limitations reduce throughput and increase cost, thereby constraining large-scale implementation [115,116]. Bridging this gap requires a stepwise transition from laboratory validation to pilot-scale verification and ultimately to commercial production, with each phase presenting distinct technical and economic challenges.

Future progress should integrate materials selection, process intensification, and manufacturing control. More sustainable CS supply chains, together with advanced EO extraction technologies, may improve resource efficiency and reduce production burdens. Reviews on postharvest coating preservation of fruits further indicate that CS and its derivatives already have a substantial application basis in fresh-produce protection, which may facilitate downstream translation once nano-enabled active systems are sufficiently standardized [117]. On the processing side, scalable fabrication routes such as intensified electrospinning, continuous emulsification, and additive manufacturing deserve greater attention. In parallel, the development of CS/nano-lignocellulose composite films for preserving cherry tomato and blueberry suggests that agricultural by-products can also be incorporated into CS-based packaging design, creating additional opportunities for resource valorization and cost-aware material development [118]. Importantly, future studies should also include techno-economic analysis, life-cycle assessment, and compatibility with existing packaging lines rather than focusing only on laboratory-scale functionality [119,120,121].

### 6.6. Consumer Acceptance and Market Adoption

Consumer acceptance will strongly influence market adoption, regardless of technical performance. Although CS-EO nano-enabled smart packaging systems may appeal to consumers because of their biodegradable matrix, natural actives, and freshness-monitoring capability, acceptance may decline if EO release alters product aroma or if nano-enabled design is perceived as unfamiliar or risky. Commercialization therefore requires not only functional efficacy, but also minimal sensory interference, transparent communication, and easily interpretable quality information [98,122].

Future research should examine willingness to pay, perceived safety, and acceptance across different food categories and consumer groups. Packaging design should likewise become more consumer-centered by incorporating intuitive visual indicators, traceability tools, and accessible digital information. In this way, trust can be built through both performance and communication, which will be essential for wider adoption of CS-EO nano-enabled smart packaging systems in real markets [96,122].

## 7. Conclusions

CS-EO nano-enabled systems represent a versatile material platform for active and smart food packaging. By using CS as a film-forming matrix and nano-delivery medium, these systems can improve the stability, dispersibility, and release control of EOs while also modulating film microstructure and, consequently, mechanical performance, barrier properties, and thermal behavior. In practical applications, CS-EO systems have shown potential for both freshness indication and responsive preservation. However, their broader implementation remains constrained by the need for long-term safety evaluation, regulatory clarity, manufacturing scalability, and consumer acceptance. Future work should therefore move beyond formulation-level optimization toward application-oriented design, with greater emphasis on standardized evaluation, scalable processing, food-specific performance validation, and improved integration of sensing, release control, and data-assisted monitoring strategies. Overall, CS-EO nano-enabled systems provide a promising route for linking functional material design with practical food-preservation needs and may contribute to the development of safer, smarter, and more sustainable packaging solutions.

## Figures and Tables

**Table 1:** Representative structural platforms for essential oil nano-delivery in chitosan-based active food packaging.

Platform	Carrier Material	Essential Oils	EE (%)	Size (nm)	Structural Feature	Suitable Food	Strength	Scale-Up Limitation	Ref.
NFs	CS/ PVA	Lemon EOs	85.56	230 ± 21.69	Dry, porous structures; highly porous 3D network; high specific surface area.	Chilled fresh meat, fresh fruits and vegetables	High kinetic stability, transparency, enhanced solubility and bioavailability, protection against oxidation and volatilization	High equipment cost, low production rate, and poor product stability.	[29]
		Lime EOs	87.16	195.12 ± 72.08					
		Grapefruit EOs	83.95	220 ± 22.58					
	CS/ Gelatin	Eucalyptus EOs	/	260.39 ± 5.05					[30]
	CS/ Gelatin	Carvacrol	/	/					[31]
NGs	CS	cinnamaldehyde EOs	83.14 ± 3.34%	421.6	Nanoscale crosslinked hydrophilic polymer network	Cheese; hydrated foods	high water content; biocompatibility; stimuli-responsive release; stable and protective.	High production cost; complex preparation; significant batch variability	[32]
	CS-caffeic acid	Zataria multiflora EOs	82.69 ± 3.12%	/					[33]
	Carboxymethyl CS, Sodium alginate	Carvacrol	88.30 ± 1.46	/					[34]
NPs	CS	Tea tree EOs	Nanospheres: 80.94%	340.07 ± 22.45	Core-shell structure	Mini cucumbers; meat emulsions	Improves solubility; increases bioavailability; slow release	Carrier materials are not reusable	[35]
	CS	curry leaf EOs	Nanospheres: 66.91%	547.9 ± 8.8					[36]
			Nanocapsules: 61.39%	640.9 ± 18.1					[36]
NEs	CS	Oregano EOs	/	144.3 ± 5.62	Nanoscale droplets (20–200 nm)	Fruits; meat; salmon	Stable, transparent	Difficult to control particle size, droplet stability, and batch reproducibility	[39]
	CS	Eryngium campestre EOs	81%	75					[40]
	CS	Clove EOs	/	45.28					[38]
	Curdlan/CS	Lavender EOs	/	152.3					[41]
PEs	Potato protein-CS NCs	Zanthoxylum bungeanum EO	92.91 ± 0.89%	280	Solid particles form a protective layer at the oil-water interface	Mandarins; blueberries; sausages	Free of surfactants, exhibiting high stability	High preparation cost; poor long-term stability	[43]
	CS NCs	Clove EOs	98.99%	180.72					[3]
	Zein-quercetin, quaternized CS	Oregano EOs	83.59%	900					[45]
NCs	CS/poly (vinyl alcohol), TiO_2_	trans-cinnamaldehyde	/	/	Uniform porous structure	Reinforced active films	Excellent bioactivity and stability	Bioaccumulation and environmental risks	[50]
	CS, TiO_2_	Daisy EOs	/	/					[51]
	CS, TiO_2_	Perilla EOs	/	D_4_,_3_: 2.87 D_3_,_2_: 1.98					[49]

Abbreviations: CS, chitosan; EO, essential oil; NF, nanofiber; NG, nanogel; NP, nanoparticle; NE, nanoemulsion; PE, Pickering emulsion; NC, nanocomposite.

**Table 2:** Main structure-property responses of essential oil nano-delivery in chitosan-based packaging films.

Property	Moderate Loading	Representative Evidence	Excess Loading	Representative Evidence	Ref.
Microstructure	Pores, cavities, cracks, phase separation	CEO/collagen-CS, 0.2%: smoothest morphology; PE/zein-CS, 1.0%: compact structure	Pores, cavities, cracks, particles	CEO/collagen-CS, 0.3–0.4%: grains/concavities; PE/zein-CS, 1.5%: particles/cracks/cavities	[51,52,55,56,57,58,59]
Thickness	Slight increase or stable	Alginate-CS/EO: 37.7–38.2 μm	Irregular variation from disrupted packing	PE/zein-CS, 0–1.5%: 0.073 → 0.091 mm	[53,57,58]
Optical properties	Lower transparency; stronger UV shielding	CEO-NE/CS, 0.6%: opacity ↑; DEO/nano-TiO_2_-CS: UV absorbance ↑	Strong scattering; poorer visual	CEO-NE/CS, higher loading: L* ↓, ΔE ↑	[38,51,59,60]
Water solubility/moisture content	Reduced	CLEO/starch-CS: MC 13.35 → 10.01%; WS 31.77 → 26.25%	Re-increase after defect formation	PE/zein-CS, 1.0%: MC 16.18%, WS 10.92%; 1.5%: both ↑	[38,55,57]
Barrier properties	Improved WVP/oxygen barrier	PE/zein-CS, 1.0%: WVP 0.33 → 0.21; CEO-NE/CS, 0.6%: OP ↓	Barrier weakening due to discontinuity	PE/zein-CS, 1.5%: WVP 0.29	[38,57,63]
Mechanical properties	Reinforcement or controlled plasticization	PE/zein-CS, 1.0%: TS 13.40 → 25.49 MPa; CEO-ME/CS, 0.6%: EAB 45.06%	Strength loss and integrity decline	PE/zein-CS, 1.5%: TS 19.51 MPa; CEO-ME/CS, higher loading: EAB 30.51%	[38,56,57]
Thermal stability	Improved	EO-NE/CS, 5%: thermal stability ↑; OEO coaxial NFs: degradation temperature ↑	Reduced after aggregation	Cinnamon EO-NE/CS, 10–15%: thermal stability ↓	[52,62,64]

Abbreviations: CEO, cinnamon essential oil; CLEO, curry leaf essential oil; DEO, daisy essential oil; NE, nanoemulsion; ME, microemulsion; PE, Pickering emulsion; TS, tensile strength; EAB, elongation at break; WVP, water vapor permeability; OP, oxygen permeability. Arrows indicate trends compared to the control: ↑ increase, ↓ decrease. The right arrow (→) indicates a change from one value to another.

**Table 3:** Representative freshness-indicating CS-EO systems classified by configuration and signal-integration mode.

System Category	Representative System	Indicator Component	EO-Related Active Component	Freshness-Indicating	Antimicrobial/Preservation	Ref.
Single-phase integrated systems	CS-OEO-BRBAII film	BRBA	OEO	pH/NH3 response; 12 d at 4 °C; red → green	87.03% (*S. aureus*); 71.01% (*E. coli*); TVB-N limit: d6 → d9	[78]
	AM/CPC/9%SFW/1.5%OC hybrid film	CPC	OC	pH/NH3 response; ΔE 23.27 (chicken), ΔE 27.04 (shrimp)	37.33 mm (*S. aureus*); 15.67 mm (*E. coli*); 33 h/21 h at 25 °C	[79]
Layered or spatially separated systems	ZN/GA/CA@PVA/CS/BBA bilayer film	BBA	CA	NH3 LOD 0.03357 μM; ΔE 2.43 → 12.16; TVB-N 8.06 → 21.82 mg/100 g	Shelf life +24 h at 4 °C	[73]
	SL-CCT bilayer film	AL	CIN-NE	Rapid, reversible pH/NH3 response; visible color change; ΔE > 5	>99% antimicrobial effect; TVB-N 22.05 mg/100 g at 48 h	[72]
Multi-signal or multimodal systems	Dual-sensor label + CS/YEO coating	BCP + MR	YEO	Fresh/semi-fresh/spoiled discrimination by dual-sensor color change	TVB-N 12.55–15.36 mg/100 g; 3.97–4.65 log CFU/g	[76]
	CS/Cur-β-CD/CIN-Z/AL double-layer film	AL + Cur	CIN	ΔE ≥ 12 or fluorescence intensity ≤ 2160	96.2% (*S. aureus*); 85.78% (*E. coli*); shelf life +2 d	[77]

Abbreviations: BRBA, black rice bran anthocyanin; OEO, oregano essential oil; CPC, Chinese purple cabbage anthocyanin; OC, Origanum compactum essential oil; BBA, blueberry anthocyanin; CA, carvacrol; AL, alizarin; CIN-NE, cinnamaldehyde nanoemulsion; BCP, bromocresol purple; MR, methyl red; YEO, yarrow essential oil; Cur-β-CD, curcumin-β-cyclodextrin complex; CIN, cinnamaldehyde; TVB-N, total volatile basic nitrogen. The right arrow (→) indicates a change from one value to another.

**Table 4:** Representative stimuli-responsive CS-EO systems showing trigger-induced release and preservation performance.

Trigger Type	Representative System	EO-Related Active Component	Trigger-Responsive	Preservation	Ref.
pH-responsive	CS-fucoidan/CIN film	CIN	Release ↑ at low pH	Litchi: ≥8 d quality	[80]
	CS-CIN Schiff-base film	CIN	pH-triggered reversible release	Mutton: +4 d at 4 °C	[81]
	CMCS-CIN emulsion gel	CIN	Acid-triggered release	Fruit preservation (on-demand)	[82]
Humidity-responsive	CS/PVA-CIN@HNT membrane	CIN	RH ↑ → release ↑	Strawberry: +150% shelf life	[86]
	CMCS/gelatin-TEO pad	TEO	10.01% → 55.59% release (30–90% RH)	Blueberry: 20 d vs. 5 d	[85]
	CS-based antimicrobial aerogel	CIN	Moisture-triggered release	Fresh-produce preservation	[87]
Temperature-responsive	Dual-sensor label + CS/YEO coating	trans-2-hexenal	Temperature-triggered release	Peach preservation	[88]
	CS-g-PNIPAm@MO microcapsule	Mugwort oil	79.2–96.1% (20–60 °C)	Sustained antibacterial	[89]
Enzyme-responsive	Gelatin-CS EO microcapsule pad	CIN + OEO	Gelatinase-triggered release	Pork: +8 d at 4 °C	[93]
Multi-stimuli-responsive	Electrospun membrane	Thymol	pH + enzyme-triggered release	pH + enzyme-triggered release	[92]
	CS/PVA nanofiber film	CIN	Antifungal (*R. stolonifer*)	Strawberry preservation	[94]

Abbreviations: CIN, cinnamaldehyde; OEO, oregano essential oil; TEO, Thyme essential oil; YEO, yarrow essential oil; HNT, halloysite nanotubes; CMCS, carboxymethyl chitosan; RH, relative humidity; PNIPAm, poly(N-isopropylacrylamide). Arrows indicate trends compared to the control: ↑ increase. The right arrow (→) indicates a change from one value to another.

## Data Availability

No new data were created or analyzed in this study. Data sharing is not applicable to this article.
